# The Effect of an App-Based Home Exercise Program on Self-reported Pain Intensity in Unspecific and Degenerative Back Pain: Pragmatic Open-label Randomized Controlled Trial

**DOI:** 10.2196/41899

**Published:** 2022-10-28

**Authors:** Hannes Weise, Benedikt Zenner, Bettina Schmiedchen, Leo Benning, Michael Bulitta, Daniel Schmitz, Kuno Weise

**Affiliations:** 1 Institute for Occupational Medicine, Social Medicine and Health Services Research University Hospital Tübingen Eberhard-Karls-University Tübingen Tübingen Germany; 2 Medical Assessment Institute Tübingen Tübingen Germany; 3 Faculty of Medicine University Hospital Tübingen Eberhard-Karls-University Tübingen Tübingen Germany; 4 Institute of Health Care and Public Management Hohenheim University Stuttgart Germany; 5 Vivira Health Lab Berlin Germany; 6 CRM Biometrics Rheinbach Germany; 7 Faculty of Medicine BG-Hospital Trauma Center Tübingen Eberhard-Karls-University Tübingen Tübingen Germany

**Keywords:** back pain, musculoskeletal health, primary care, exercise therapy, digital health, mobile health, mHealth, digital therapeutic, mobile phone

## Abstract

**Background:**

The recommended first-line treatment for unspecific and degenerative back pain consists of movement exercises and patient education.

**Objective:**

Using a pragmatic, randomized controlled trial, we evaluated the effectiveness of a digital home exercise program on self-reported pain intensity compared with the standard of care for physiotherapy.

**Methods:**

Participant recruitment was based on newspaper advertisements and a consecutive on-site assessment for eligibility and enrollment. Participants with unspecific and degenerative back pain aged ≥18 years were randomly assigned in a 1:1 ratio to receive a 12-week stand-alone digital home exercise program or physiotherapy. The digital home exercise program included 4 exercises daily, while physiotherapy included 6 to 12 sessions, depending on the severity of symptoms. The primary outcome was pain, which was assessed using a verbal numerical rating scale. The clinical relevance of pain reduction was assessed using the following thresholds: improvement of at least 1.4 points on the verbal numerical rating scale and a pain reduction of at least 30%.

**Results:**

During the study period, 108 participants were assigned to the intervention group and 105 participants to the control group. The mean difference in pain scores between the 2 groups at 12 weeks was −2.44 (95% CI −2.92 to −1.95; *P*<.01) in favor of the intervention group. The group receiving the digital therapeutic achieved a clinically relevant reduction in pain over the course of the study (baseline vs 12 weeks), with a mean change of −3.35 (SD 2.05) score points or −53.1% (SD 29.5). By contrast, this change did not reach clinical relevance in the control group (mean −0.91, SD 1.5; −14.6%, SD 25.3). Retention rates of 89.9% in the intervention group and 97.3% in the control group were maintained throughout the study.

**Conclusions:**

The use of the app-based home exercise program led to a significant and clinically relevant reduction in pain intensity throughout the 12-week duration of the program. The intervention studied showed superior improvement in self-reported pain intensity when compared with the standard of care. Given the great demand for standard physiotherapy for unspecific and degenerative back pain, digital therapeutics are evolving into a suitable therapeutic option that can overcome the limitations of access and availability of conventional modes of health care delivery into this spectrum of indications. However, further independent evaluations are required to support the growing body of evidence on the effectiveness of digital therapeutics in real-world care settings.

**Trial Registration:**

German Clinical Trials Register DRKS00022781; https://tinyurl.com/hpdraa89

## Introduction

Musculoskeletal conditions are among the top drivers of the burden of disease worldwide. In the most recent Global Burden of Disease Study, lower unspecific back pain accounted for 2.5% of all disability-adjusted life years [1]. Although the spectrum of musculoskeletal conditions shows a high prevalence among older individuals, it also accounts for significant direct and indirect health care expenses in other age strata [2]. Hence, health care systems face the challenge of providing adequate and timely care for these conditions. The need for adequate and comprehensive care settings has long been identified [3,4], but the availability of and access to adequate care often remains limited. For the spectrum of unspecific musculoskeletal conditions, physiotherapy and other forms of exercise-based therapies have been described as first-line treatments in international guidelines [5-7]. However, these therapies are often not sufficiently available owing to regulations in health care policy [8], limited availability of and access to care [9,10], as well as challenges regarding the delivery of care [11,12].

In this context, new and innovative approaches are required to develop and sustain a responsive and accessible health care delivery infrastructure. While numerous attempts have been made to digitize components of health care related to musculoskeletal conditions, most have failed to be integrated into existing health care systems and established care delivery pathways [13]. However, after the introduction of the Digital Health Care Act (Digitale-Versorgung-Gesetz) in Germany in 2019, digital health apps, referred to as Digitale Gesundheitsanwendungen (DiGA), were established as a new category of digital therapeutics. These digital therapeutics could receive full market approval from the Bundesinstitut für Arzneimittel und Medizinprodukte (BfArM), the German body that assesses pharmaceutical products and medical devices regarding their safety and effectiveness and grants market approval. Since then, approved DiGA have become part of the collectively funded health insurance system and can be prescribed by all licensed physicians and other health care professionals in Germany.

In view of this, ViViRA (ViViRA Health Lab GmbH) is the first self-guided home exercise program for the treatment of degenerative and unspecific back pain that has been approved by the BfArM for use in the collectively funded statutory health insurance system. Thus, ViViRA can be integrated into routine medical care in Germany. For approval as a DiGA by the regulatory bodies, a randomized controlled trial demonstrating effectiveness was conducted. Hence, this publication presents data from a pragmatic, open-label randomized controlled trial that aimed to assess the comparative effectiveness of BfArM-approved DiGA ViViRA against the established standard of care for physiotherapy.

## Methods

### Trial Design

We conducted a pragmatic randomized controlled trial with 1 intervention group and 1 control group. As the mode of administration of the experimental therapy (ie, in the intervention group) differed significantly from that of the control therapy (ie, in the control group), an open-label design was chosen. The intervention group used the digital therapeutic ViViRA, while the control group received the standard treatment of physiotherapy. Intervention and control therapies were administered in parallel. All study-related data (ie, baseline assessment, primary end point data, and supplementary data) were collected between August 2020 and April 2021. No modifications were made to the trial design after its commencement.

### Study Population

Inclusion criteria and requirements for participation in the study were defined as follows: (1) age >18 years; (2) diagnosis of a unspecific or degenerative pain of the lower back (International Classification of Diseases, 10th edition M42.0, M42.1, M42.9, M53.2, M53.8, M53.9, M54.4, M54.5, M54.6, M54.8, M54.9, M99.02, M99.03, M99.04, M99.82, M99.83, M99.84, M99.92, M99.93, and M99.94); (3) a pain score of ≥4 out of 10 based on the verbal numerical rating scale (VNRS) at the time of enrollment, which corresponds to at least moderate pain and is a plausible indicator of therapeutic need in a real-world setting; (4) possession of a mobile device (ie, smartphone or tablet) and the ability to use such a device; and (5) ability to provide informed consent. The exclusion criteria are outlined in Textbox 1.

**Exclusion criteria to participate in the study.**

**General**

- No pain, pain score ≤3
- Previous movement therapy with a digital therapeutic for musculoskeletal pain
- Use of analgesics before inclusion
- Pregnancy
- Limited legal or insufficient language capacity
- Patients who are not able to follow the exercise protocol; for example, significantly impaired vision or blindness

**Internal**

- Severe organ failure
  Condition after heart attack
  Need for dialysis
  Cardiovascular decompensation
  Pulmonary insufficiency
- Inflammation
  Past or present rheumatological disease
  Acute inflammatory diseases
  Feverish condition
- Coagulopathy
  Thrombosis
  Blood coagulation disorders including anticoagulant therapy

**Musculoskeletal**

- Any bone disease
- Injuries or surgery
  Fresh bone or joint fractures
  Injury to spinal column, knee, or hip joint
  Condition after
  Spine, hip, or joint surgery
  Osteotomy (an operation to correct the axis of the leg)
  Arthrodesis (joint stiffening) in 1 of the 2 knee or hip joints
- Inflammatory disease
  Spinal column or joint inflammatory disease
  Situation after spinal column or joint inflammatory disease
- Spinal tumor
- Osteochondrosis dissecans
- Bone necrosis
- Hip dysplasia
- Acute instability of the knee or hip joint
- Free joint bodies
- Disc pathology
  Slipped disc
  Acute herniated disc or other disorder with radiation to the legs (radiculopathy or sensorimotor failure)
  Herniated disc in the past
- Clinically relevant bone marrow edema
- Osteoporosis

**Neuropsychiatric**

- Serious neurological disorders
  Stroke
  Paralysis
  Multiple sclerosis
  Convulsions
- Posture insecurity
  Neurological motor disorders
  Sensomotoric disorders
  Vertigo
- Skin sensitivity disorder
- Psychoses
- Dementia
- Drug or alcohol abuse

**Oncological**

- Metastases of malignant tumors
- Acute malignant disease

Recruitment was initiated through newspaper advertisements in 2 regions of the state of Baden-Wuerttemberg, Germany. Patients interested in participation underwent prescreening by telephone before undergoing an interview and a physical examination. Physical examinations and baseline assessments were conducted by an investigator (HW and KW) and study nurses at an outpatient study center affiliated with the University Hospital Tübingen. All follow-up assessments were conducted remotely via phone calls and questionnaires. Study nurses coordinated follow-up appointments and monitored the completion of follow-up assessments. The trial ended after 12 weeks. No reason for the early termination of the trial was reported throughout the duration of the study. Participants were not paid for trial participation; however, costs resulting directly from trial participation were reimbursed (eg, travel expenses incurred for the baseline visit).

### Intervention

**Figure 1:**
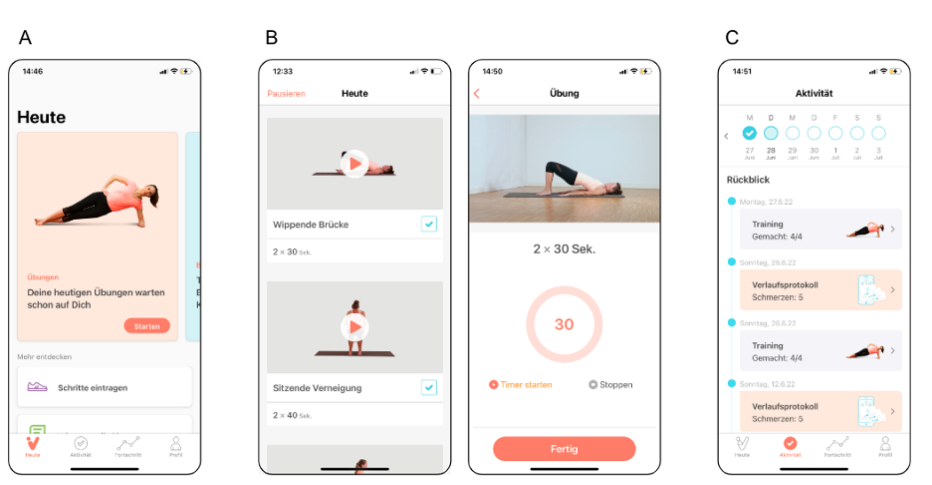
Patient interface of the digital home exercise program. (A) Home screen with daily prompt to start the exercises in the German language setting. (B) Composition of 4 exercises based on baseline assessment and patient feedback on pain and functional limitations (left); example of the video- and audio-guided exercise screen (right). (C) Summary of completed exercises, follow-up assessments, and therapeutic progress achieved.

The interventional group was provided access to the digital therapeutic on their mobile device free of charge. Patients in this group were asked to exercise at least three days per week throughout the trial period of 12 weeks, and patients were advised to use the default notification setting with a daily reminder displayed as a push notification. The digital therapeutic assessed in this trial was the ViViRA app (ViViRA Health Lab GmbH), an approved DiGA addressing the indication spectrum outlined earlier. It is a medical device used in mobile devices with iOS and Android operating systems, providing a self-directed home exercise program using the principles of movement therapy and functional regional interdependence, as outlined elsewhere [7,14-16]. The intervention is only available through a prescription or an individual subscription. No updates or changes were made to the therapeutic elements of the app during the duration of the study. All patients in the investigational group were provided the same version of the app. The user interface and the prompt of an example exercise is displayed in Figure 1. Patients were prompted to complete 4 exercises per day for 12 consecutive weeks. Guidance on how to exercise is given multimodally using demonstration videos as well as written and audio instructions. After each exercise, patients provide feedback that allows for the continuous adoption of exercise selection based on pain and physical ability. A progression algorithm modifies exercise composition, exercise intensity, and exercise complexity. The development of the progression algorithm was led by an interdisciplinary expert panel consisting of orthopedic surgeons and physiotherapists. The control group received treatment in line with German treatment guidelines [14,17] recommending physiotherapy. This includes physical exercises lasting 15 to 25 minutes guided by a certified physiotherapist. According to the German treatment guidelines [17], treatment includes 6 to 12 such physiotherapy sessions for each prescription. Patients in the control group were assigned to receive physical therapy from a certified physiotherapist of their choice. No influence was exerted on therapist choice, scheduling, waiting times, or additional physiotherapy sessions; however, any costs incurred were covered by the sponsor of the trial.

Adherence to self-directed exercise therapy was assumed if at least one training was finished per week, and the patient confirmed within the app that they had done the exercises by providing feedback on the feasibility of each exercise and any pain experienced during the exercises. Information on adherence to the standard of care was obtained during follow-up interviews.

### Outcome Measure

Self-reported pain intensity was assessed as the primary outcome measure using a VNRS that was linguistically adapted for German-speaking study participants. A nonequidistant scaling of pain score categories across an 11-point rating scale from 0 to 10 (corresponding to 0-100 mm on a visual analog scale) was used [18]. An adaptation to the proposed scale was made according to Weber et al [19] as the 2 categories for highest pain were integrated. The primary outcome was assessed at baseline and after 2, 6, and 12 weeks in both the control and intervention groups. Secondary analysis of total pain scores and their changes during the study were determined a posteriori. No changes were made to the outcome of the study after it had commenced.

### Assessment of Potential Harms

An active surveillance of adverse events (AEs) and unintended effects in both the intervention and control groups was conducted during structured interviews at weeks 2, 6, and 12 after the baseline examination. A differentiation of AE and adverse reactions (ARs) to either the administration of the intervention or control exercise therapy was conducted accordingly.

### Sample Size

To determine the required sample size, a trial with a 2-sided question (significance level Cronbach α=5%; power 1-β=80%) was planned. We used retrospective pilot data from patients who had been applying the digital home exercise program between 2018 and 2019. According to the pilot data, the VNRS limit was 1 score point and the approximate SD was 2.5 score points. This produced a standardized delta of Δ=1/2.5=0.4. Calculations with a significance level of Cronbach α=5% and a power of 1-β=80% resulted in 2×99 patients (N=198). To account for a potential dropout to study surveys of approximately 10%, we included 213 patients in this study, with 108 (50.7%) and 105 (49.3%) randomized to the investigation and control groups, respectively. This study was designed to assess the superiority of the intervention against the standard of care treatment.

### Randomization

Participants who met the inclusion criteria outlined earlier were randomly assigned to either the investigation or control group. Randomization was based on block randomization with a block size of 6, generated with the SAS module *Proc Plan* The allocation ratio at the time of randomization was 1:1. The randomization list was generated by the data manager of the Contract Research Organization CRM Biometrics GmbH (DS). On entry into the study, each patient was assigned a patient identification number. Using the patient identification number, each included patient was assigned to either the intervention or control group in the sequence specified by the randomization blocks. No deviation from the randomization sequence was reported.

### Statistical Analysis

Data analysis was performed by a biostatistician who was not involved in the collection of the analyzed data. Analyses were performed according to the intention-to-treat (ITT) approach. Data from participants with pain scores based on the VNRS at baseline were used in this analysis. It included all participants who were randomized and showed values for the primary variable at baseline. Furthermore, we conducted the same analyses in the prespecified per-protocol (PP) set. This included patients who were not lost to follow-up. In addition, patients who stated at the follow-up assessments that they had received concomitant physiotherapy or taken pain medication during the intervention period were excluded from the PP analysis of the intervention group. Similarly, in the control group, patients who reported concomitant use of pain medications or of a home exercise program during the study period were excluded. Metric data are expressed as means with SDs or 95% CIs. Nominal (sex) and ordinal (shift of pain score) data are reported as the cell frequencies and percentages of patients in each category. Between-group and intragroup differences were calculated using Welch 2-tailed *t* test. Score differences and Cohen delta were calculated for confirmatory treatment group comparisons (intervention vs control group), as well as for intragroup score changes from baseline to follow-ups. Cohen *d* was used for a quantitative and metric-free estimation of the effect size, with values >0.20 defined as small effect sizes, >0.50 as medium effect sizes, and >0.80 as large effect sizes [20]. All hypothesis tests used were 2-sided, and *P*≤.05 was considered significant. Statistical analyses were performed with SAS, version 94M7 (SAS), and GraphPad Prism, version 9.1.0 (GraphPad).

### Ethics Approval

The study concept was reviewed and approved by the Institutional Ethics Commission of the Chamber of Physicians and Surgeons of the State of Baden-Wuerttemberg, Germany, under the registration number F-2020-122 and in agreement with current data protection regulations. The trial is registered at Deutsches Register Klinische Studien (Germany Clinical Trials Register; World Health Organization Primary Register) with the identifier DRKS00022781. Before enrollment in the study, patients received oral information from a trial physician and written patient information that included a description and purpose of the study, possible AEs, the name and address of the insurer, and information on data protection. Thereafter, the patients signed a written informed consent form to participate in the study and consented to the use of their data. This manuscript was prepared in accordance with the 2010 CONSORT (Consolidated Standards of Reporting Trials) guidelines. The intervention studied is outlined in detail in the attached TiDier (template for intervention description and replication) checklist (Multimedia Appendix 1).

## Results

### Included Patients

**Figure 2:**
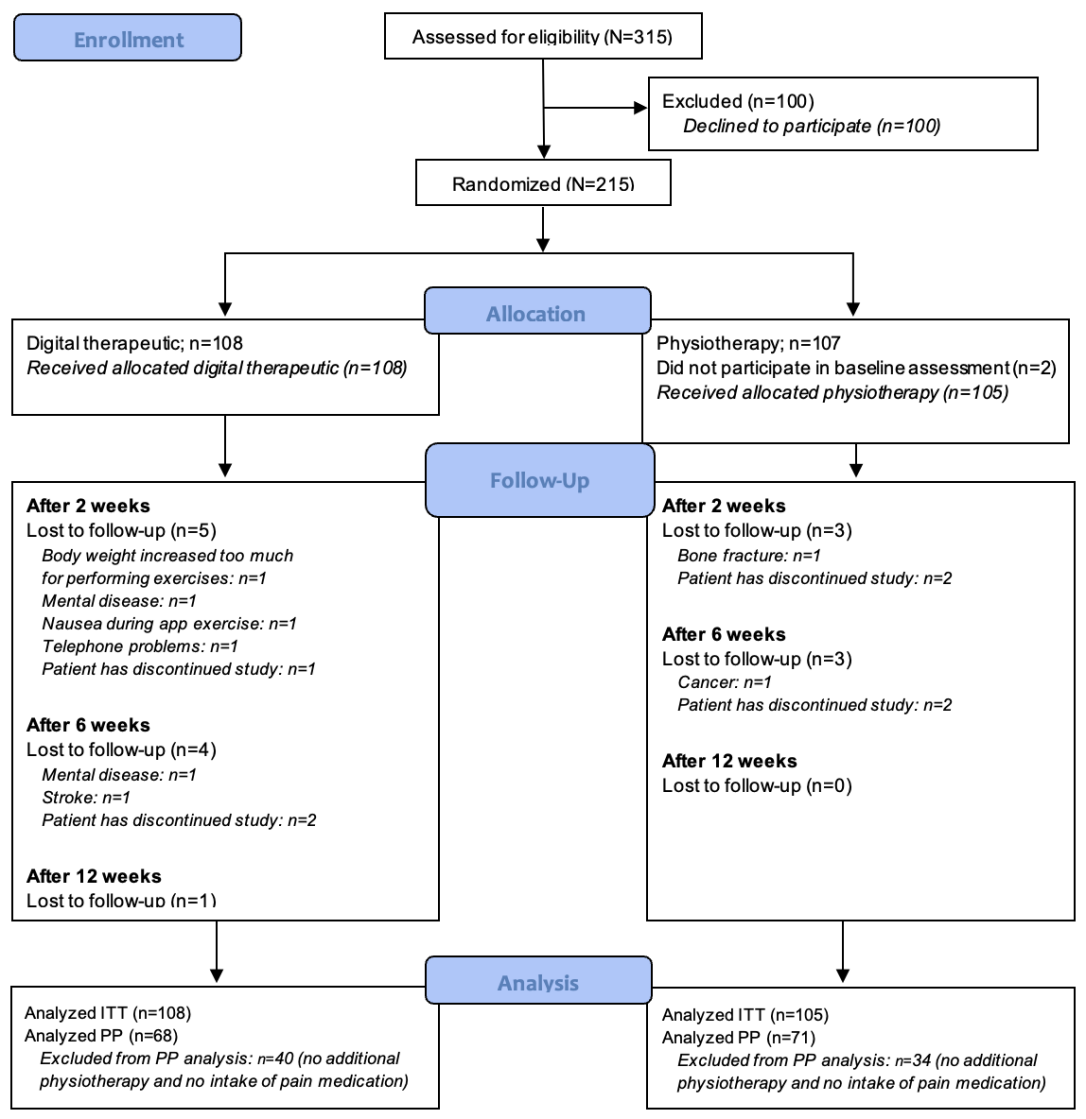
CONSORT (Consolidated Standards of Reporting Trials) flowchart on screening, inclusion, randomization, follow-up, and analysis. ITT: intention-to-treat; PP: per-protocol.

A total of 215 patients were enrolled and randomly allocated to the intervention (n=108) and control (n=107) groups. In total, 2 patients in the control group did not respond to the baseline follow-up call and did not provide any outcome data after randomization. Therefore, these patients were considered screening failures and were not included in the subsequent analysis. This reduced the total number of patients in the control group to 105. No violations of the protocol were reported, which would have led to an exclusion from the study. All patients enrolled and randomly allocated were included in the ITT analysis. For the PP analysis, 68 patients of the intervention group and 71 patients of the control group were considered. Figure 2 displays the follow-up chart.

### Recruitment

The recruitment period, as outlined in the Methods section, started in August 2020. The final follow-up was completed by the end of April 2021. The baseline assessment was conducted onsite, whereas follow-up assessments after 2, 6, and 12 weeks were conducted remotely via phone calls and questionnaires. The trial ended after 12 weeks. No reason for an early termination of the trial was reported during the 12-week study period. Table 1 presents the demographic and clinical characteristics of the study participants at the baseline.

**Table 1:** Baseline demographic and clinical characteristics of the intention-to-treat population (N=213).

		Intervention group^a^	Control group^b^
Participants, n (%)		108 (50.7)	105 (49.3)
Age (years), mean (SD)		57.4 (13.8)	57.3 (13.5)
Sex (female), n (%)		51 (47.2)	62 (59.1)
**Indications (ICD-10^c^), n (%)**			
	M54.4—lumbago with sciatica	44 (40.7)	33 (31.4)
	M54.5—low back pain	44 (40.7)	45 (42.9)
	M54.9—dorsalgia, unspecified	20 (18.5)	27 (25.7)
Pain score (VNRS^d^ 0-10), mean (SD)		6.41 (1.65)	6.05 (1.64)

^a^Patients in the intervention group used a digital home exercise program to treat their back pain.

^b^Patients in the control group received the standard of care (ie, physiotherapy).

^c^ICD-10: International Statistical Classification of Diseases, 10th edition.

^d^VNRS: verbal numerical rating scale.

### Primary Outcome

#### Intragroup Comparison

Pain responses were assessed with a German VNRS validated for nonmalignant pain [18]. All patients were analyzed in the group they were initially assigned to (ie, ITT analysis). From a mean baseline pain score of 6.42 (SD 1.65), the intervention group with 108 participants showed a significant reduction of the pain score to 3.94 (SD 1.79) after 2 weeks to 3.50 (SD 2.21) after 6 weeks and to 3.06 (SD 2.18) after 12 weeks of exercise therapy. These changes are of statistical significance as compared with the assessed baseline pain score (all *P*<.001; Cohen *d*>0.8). Comparing the mean pain scores at baseline and after 12 weeks, the perceived pain decreased by a mean of −3.35 (SD 2.05) score points and −53.1% (SD 29.5%; Table 2). These significantly lower values of reported pain scores at week 12 as compared with the baseline assessment were also found in the PP analysis (*P*<.001).

**Figure 3:**
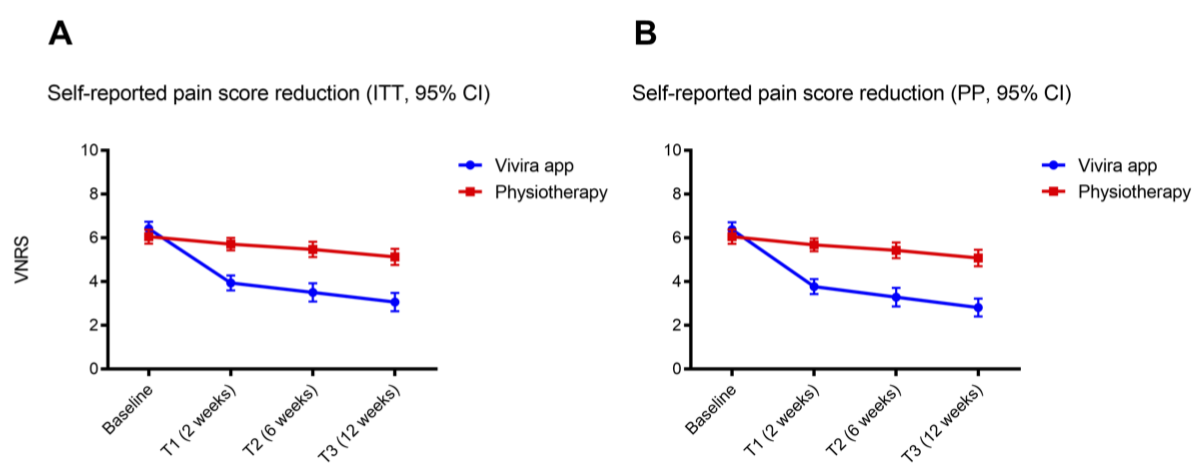
Pain score values assessed by the verbal numerical rating scale (VNRS) in the (A) intention-to-treat (ITT) and (B) per-protocol (PP) populations at baseline and after 2, 6, and 12 weeks of intervention. Dot plots showing mean pain score values assessed by the VNRS in the patients receiving the digital therapeutic (investigation group, blue) and the conventional physiotherapy (control group, red) of the (A) ITT and (B) PP populations. Error bars indicate 95% CIs.

The control group of 105 participants reported a reduction in pain to a lesser extent as compared with the intervention group. From a reported mean baseline of 6.05 (SD 1.64), a marginal reduction to 5.71 (SD 1.48; *P*=.123; Cohen *d*=0.22) could be observed after 2 weeks. After 6 and 12 weeks, significant pain score reductions to 5.47 (SD 1.80; *P*<.05; Cohen *d*=0.34) and 5.13 (SD 1.91; *P*<.001; Cohen *d*=0.52), respectively, were observed (Figure 3). This reduction in pain corresponds to a mean reduction in perceived pain by −0.91 (SD 1.50) score points and by −14.6% (SD 25.3%; Table 2). Regarding the PP analysis, the described pain reduced significantly (*P*<.001).

**Table 2:** Absolute and relative pain score (VNRS^a^) changes after 2, 6, and 12 weeks of the intention-to-treat population.

			Intervention group (n=108)							Control group (n=105)				
			2 weeks after baseline		6 weeks after baseline		12 weeks after baseline		2 weeks after baseline			6 weeks after baseline		12 weeks after baseline
**Absolute pain score (VNRS) change,** **mean (SD)**			−2.47 (1.74)		−2.92 (2.07)		−3.35 (2.05)		−0.33 (1.42)			−0.58 (1.65)		−0.91 (1.50)
	Cohen *d*, between-group comparison^b^	1.35		1.26		1.37		N/A^c^			N/A		N/A	
	*P* value^d^, between-group comparison	<.001		<.001		<.001		N/A			N/A		N/A	
**Relative pain score change (%),** **mean (SD)**			−38.0 (22.9)		−45.7 (30.6)		−53.1 (29.5)		−2.45 (24.2)			−7.14 (28.3)		−14.6 (25.3)
	Cohen *d*, between-group comparison	1.51		1.31		1.40		N/A			N/A		N/A	
	*P* value, between-group comparison	<.001		<.001		<.001		N/A			N/A		N/A	

^a^VNRS: verbal numerical rating scale.

^b^The statistical comparison of the between-group differences was calculated using the 2-tailed *t* test.

^c^N/A: not applicable.

^d^A *P* value of <.05 was considered statistically significant.

#### Between-Group Comparison

Participants of the intervention group reported significantly lower pain intensity than those of the control group from 2 weeks after the start of the study (Figure 2). Between-group differences in reported pain scores showed significantly greater improvements in the intervention group at week 2 (−2.12, 95% CI −2.57 to −1.71; *P*<.01), week 6 (−2.34, 95% CI −2.84 to −1.83; *P*<.01), and week 12 (−2.44, 95% CI −2.92 to −1.95; *P*<.01; Table 3) after the baseline assessment. These results are consistent with those of the PP analysis, where the mean reported pain score was significantly lower in the intervention group as compared with the control group (each *P*<.001; Multimedia Appendix 2).

**Table 3:** Between-group differences^a^ of absolute pain score (verbal numerical rating scale) after 2, 6, and 12 weeks of the intention-to-treat population.

Absolute pain score changes (score points)	Week 2	Week 6	Week 12
Mean	−2.14	−2.34	−2.44
95% CI	−2.57 to −1.71	−2.84 to −1.83	−2.92 to −1.95
*P* value^b^	<.001	<.001	<.001

^a^The statistical comparison of the between-group differences was calculated using the 2-tailed *t* test.

^b^A *P* value of <.05 was considered statistically significant.

### Secondary Analysis of the Overall Pain Scores

A secondary analysis of the pain scores revealed substantially fewer pain exacerbations among the participants of the intervention group as compared with the control group, that is, an increase in pain intensity compared with the reported pain score at baseline.

In the intervention group, 2.77% (3/108) of the patients reported an increase in perceived pain after 6 weeks of treatment but fully recovered after the full duration of the 12-week exercise training program. However, most patients of this group (99/108, 91.7%) reported a reduction in perceived pain.

By contrast, in the control group, 22.9% (24/105) of the patients experienced an increase in pain intensity after 2 weeks and 24.8% (26/105) of the patients after 6 weeks. The number of patients of the control group who reported an increase in pain decreased marginally to 17.1% (18/105) after 12 weeks. However, 60% of the patients of this group reported an improvement in perceived pain (Table 4).

**Table 4:** Shift of the pain score from baseline to weeks 2, 6, and 12 within the intention-to-treat population.

	Intervention group, VNRS^a^ pain score shift			Control group, VNRS pain score shift		
	Improved	No change	Exacerbation	Improved	No change	Exacerbation
Week 2, n (%)	97 (89.8)	11 (10.2)	0 (0)	42 (40)	39 (37.1)	24 (22.9)
Week 6, n (%)	95 (87.9)	19 (9.3)	3 (2.8)	50 (47.6)	29 (27.6)	26 (24.8)
Week 12, n (%)	99 (91.7)	9 (8.3)	0 (0)	63 (60.0)	24 (22.9)	18 (17.1)

^a^VNRS: verbal numerical rating scale.

### Adherence

Patients in the intervention group of the ITT cohort were using the exercise therapy on an average of 5.77 days out of 7 possible days per week. This corresponds to an adherence rate of 89.9%. The patients of the control group received a mean of 6.94 (SD 2.94) physiotherapy sessions during the 12-week study period. Adherence in the control group was defined as the percentage of 695 physiotherapy sessions completed versus the planned number of 714 sessions, resulting in an adherence rate of 97.3%.

### Adverse Reactions and Adverse Events

ARs were reported by 34.3% (37/108) of the patients in the intervention group and 29.5% (31/105) of the patients in the control group and are outlined in Multimedia Appendix 3. None of the reported ARs led to the discontinuation of the intervention in either group. In addition, no serious AEs were reported. No privacy breaches or substantial technical problems were detected.

## Discussion

### Principal Findings

This study aimed to assess the comparative effectiveness of a digital home exercise program and the standard of care treatment for unspecific and degenerative back pain. The results of the present study show that the use of a digital home exercise program can lead to a significant and clinically relevant reduction in patient-reported unspecific and degenerative back pain. Moreover, the results of the present study indicate that the reduction of self-reported pain intensity achievable with the digital therapeutic under investigation is superior to the reduction of self-reported pain intensity achieved with the standard of care (mean difference of the assessed pain score at 12 weeks: −2.44, 95% CI −2.92 to −1.95, in favor of the intervention group).

### Limitations

The data presented in this paper contribute to the growing body of knowledge in the field of digital therapeutic interventions. Through a pragmatic randomized controlled design, this trial aimed to substantiate the evidence for the effectiveness of the digital home exercise program ViViRA. Nonetheless, we see factors that limit the external validity of our study and thus the generalizability of our findings.

First, the decentralized nature of digital therapeutics is a key factor leading to better access to and availability of therapeutic resources compared with physiotherapy treatments in a physiotherapist’s practice. This comes at the expense of a close interpersonal relationship between patients and health care professionals, which naturally contributes to the effectiveness of therapeutic interventions [21,22]. However, as this study relied on the conventional (ie, out of app) collection of data through phone calls and questionnaires (as compared with in-app and real-world data analyses), the trial staff maintained close contact with the enrolled patients. Therefore, a potential observer bias as well as a detection bias needs to be taken into account when applying these results to a real-world use scenario, and further research on observational or real-world use data is required to assess the extent of these potential biases.

Second, the enrollment for the trial presented was primarily based on newspaper advertisements in 2 regions in the German federal state of Baden-Wuerttemberg. As this differs from the enrollment in clinical practice, a selection bias is plausibly present in this study, as a DiGA typically requires a prescription from a health care professional (ie, provider-driven initiation of therapy) and is only very limited accessible through self-selection (ie, patient-driven initiation of therapy). As discussed earlier, more research on the relevance of these differential patient motivations is required.

Third, the availability of physiotherapists is limited and varies by region. Therefore, system-related waiting times are likely to reduce the therapeutic density (ie, the number of therapeutic sessions per week), which affects the expected effectiveness in the control group. This difference in effectiveness between the intervention and the standard of care is likely to be emphasized by the decentralized and on-demand availability of digital therapeutics. This could explain the small effect of physiotherapy on pain intensity in comparison with the intervention examined. However, given that improved access to and availability of digital therapeutics are key characteristics of digital therapeutics, we considered the comparison appropriate in the context of real-world use.

Further limitations of this study include the nonblinded design, which was required as the mode of administration of the intervention and the control differed significantly and could not be feasibly blinded, and the lack of an objective measure for perceived pain intensity, which is challenging because of its highly individual nature. Generally, self-reported pain intensity is considered to be validly measured by different pain scales. This study relied on a German VNRS that has been validated for nonmalignant pain [18]. In addition, sufficient comparability with other unidimensional pain intensity scales has been demonstrated by other researchers [23,24].

### Comparison With Prior Work

This study focused on assessing the effectiveness of digital therapy compared with physiotherapy, the standard of care. During the 12-week exercise program with digital therapeutic, 91.7% (99/108) of the patients described pain relief. On the basis of the VNRS, which has been used to quantify pain, this corresponds to a mean pain relief of −52.3%. These results complement the existing literature, as comparable positive effects on pain intensity between −33.3% and −81% have been described in several studies assessing the effectiveness of digital therapeutics for musculoskeletal conditions [25-29]. As equivalent intervention periods of 12 weeks were studied, we deemed the comparison with these studies applicable. However, we deem the comparison with the work of Shebib et al [27] as particularly comparable, as this group also assessed a stand-alone intervention for the treatment of lower back pain. This group demonstrated an average pain score improvement between 52% and 64% [27]. Other works, for example, from Priebe et al [26] and Sandal et al [29], pursued an add-on approach to augment the existing infrastructure of care and can, therefore, not be considered a stand-alone intervention. Apart from the effectiveness of the digital therapeutic, it is also necessary to consider the effects that were achieved in the control group by the standard of care. Interestingly, similar to the previously mentioned studies [25-27], the extent to which physiotherapy was able to achieve a substantial reduction in pain intensity was lower as compared with digital exercise therapy. As outlined earlier, we do not interpret this as evidence for physiotherapy not being effective in reducing self-reported pain intensity but attribute this primarily to the different modes of administration. The centralized (ie, onsite) and synchronous (ie, by appointment) administration of physiotherapy limits the patient-specific adaptation of therapy intensity and frequency and, hence, can lead to suboptimal therapeutic results. We see this reflected in the average therapy frequency in our data: patients in the intervention group used the exercise training on average for 5.77 days per week. In comparison, patients in the control group received 6.94 physiotherapy sessions during the entire 12-week study period. In addition, the German health care system has reimbursement limits and provider-specific budgets for the number of physical therapy sessions available to a patient. These system-inherent limitations, from our point of view, reduce the achievable positive effects of conventional physiotherapy, as observed in this study. By contrast, no such differences or much smaller differences between a digital therapeutic and the standard of care treatment have been observed in other studies. Sandal et al [29], for example, described significant, though smaller, between-group differences in pain intensity in favor of an artificial intelligence-based app to self-management support system for treatment of lower back pain, compared with standard of care. However, as discussed earlier, the overall approach of this group differs from the assessment presented here, as Sandal et al [29] studied an add-on intervention for the treatment of lower back pain. Similarly, Koppenaal et al [30] assessed the effectiveness of blended physiotherapy (digital exercise training with face-to-face physiotherapy sessions) compared with the standard of care and found no group differences in pain reduction. However, an exception to this overall finding is the group of patients at a high risk of developing persistent low back pain in which blended therapy was superior to physiotherapy in terms of average reported pain reduction [30]. These results underscore, from our perspective, the advantages of decentralized and immediately available digital therapies for the treatment of back pain. Furthermore, Lara-Palomo et al [31] found no difference in effectiveness in reducing back pain when comparing digital health apps and standard face-to-face care in a systematic review and meta-analysis. Although the available evidence was only considered to be of moderate quality, we interpret these results as yet another indicator for the quality and effectiveness of care that can be delivered through digital therapeutics.

Finally, and especially in the case of patient-oriented outcomes, it is important to assess the clinical relevance of the results obtained. Several thresholds for assessing the clinical relevance of improvements in pain intensity have been defined in the literature. Exemplarily, and according to Ostelo et al [32], this threshold is a reduction of 30%, considered a minimally important change, while Holdgate et al [33] referred to a 1.4 score point improvement in VNRS as the minimum clinically significant difference. Applying these criteria underlines that the effect of the digital home exercise program on pain intensity was not only statistically significant but also clinically relevant for patients. The positive effect on a reduction in pain intensity to a clinically relevant extent was measurable after the second week of exercise therapy in the investigation group. Interestingly, the thresholds discussed above were not met by patients in the control group. To our knowledge, none of the previous studies with a digital therapeutic focused and described such an early effect. Before this background, digital home exercise programs can be considered a veritable therapeutic option for unspecific and degenerative back pain, which is in line with national and international treatment recommendations [6,14,15] that prioritize movement and exercise therapy over medication and more invasive therapeutic measures. In terms of potential harms associated with the use of the digital therapeutic, we noted several AEs, all of which were transient in nature (Multimedia Appendix 2). Therefore, we conclude that no intolerable risks are associated with the use of the program assessed within the scope of its approved indications and considering the exclusion criteria.

### Conclusions

In the face of an increasing burden of disease from unspecific and degenerative musculoskeletal conditions, novel and innovative therapeutic approaches are required to ensure access to and availability of effective care for this spectrum of conditions. With the introduction of the DiGA into the collectively funded German health care system, a regulatory framework for the system-wide implementation of digital therapeutics was created. This study presents effectiveness data for one of the first fully approved DiGAs and shows significant and clinically relevant improvements in self-reported pain intensity. These improvements were superior to those of the control group, representing the current standard of care in the German health care system. By expanding the available therapeutic capacities for unspecific and degenerative back pain through a decentralized and on-demand digital therapeutic, a significant added value in pain management can be achieved.

Given the high burden of disease caused by back pain and the limited availability of and access to adequate health care, digital apps are an efficient treatment option for unspecific and degenerative back pain. In view of this, replication of the present trial in further independent studies considering additional outcome parameters, such as function, with longer follow-up periods, for example, 6 and 12 months, and its applicability in other countries and health care systems is of great interest. In addition, particularly in the field of digital therapeutics, further research on available real-world use data will complement the formalized and trial-based assessments of such therapeutics. By generating an increasing body of evidence as well as integrating digital apps into health care systems, digital therapeutics can contribute significantly to health care in the indication area.

## Appendix

Multimedia Appendix 1 Template for intervention description and replication (TIDieR) checklist for the intervention studied.

Multimedia Appendix 2 Pain score values assessed by the verbal numerical rating scale in the per-protocol population at baseline and after 2, 6, and 12 weeks of intervention.

Multimedia Appendix 3 Adverse reactions reported by the study population during the 12-week intervention period.

Multimedia Appendix 4 CONSORT-eHEALTH checklist (V 1.6.1).

## Abbreviations

AE: adverse event
AR: adverse reaction
BfArM: Bundesinstitut für Arzneimittel und Medizinprodukte
CONSORT: Consolidated Standards of Reporting Trials
DiGA: Digitale Gesundheitsanwendungen
ITT: intention-to-treat
PP: per-protocol
TiDier: template for intervention description and replication
VNRS: verbal numerical rating scale
