# Correction: The Effect of an App-Based Home Exercise Program on Self-reported Pain Intensity in Unspecific and Degenerative Back Pain: Pragmatic Open-label Randomized Controlled Trial

**DOI:** 10.2196/46512

**Published:** 2023-02-20

**Authors:** Hannes Weise, Benedikt Zenner, Bettina Schmiedchen, Leo Benning, Michael Bulitta, Daniel Schmitz, Kuno Weise

**Affiliations:** 1 Institute for Occupational Medicine, Social Medicine and Health Services Research University Hospital Tübingen Eberhard-Karls-University Tübingen Tübingen Germany; 2 Medical Assessment Institute Tübingen Tübingen Germany; 3 Faculty of Medicine University Hospital Tübingen Eberhard-Karls-University Tübingen Tübingen Germany; 4 Institute of Health Care and Public Management Hohenheim University Stuttgart Germany; 5 Vivira Health Lab Berlin Germany; 6 CRM Biometrics Rheinbach Germany; 7 Faculty of Medicine BG-Hospital Trauma Center Tübingen Eberhard-Karls-University Tübingen Tübingen Germany

In “The Effect of an App-Based Home Exercise Program on Self-reported Pain Intensity in Unspecific and Degenerative Back Pain: Pragmatic Open-label Randomized Controlled Trial” (J Med Internet Res 2022;24(10):e41899) the authors made one addition.

Under Acknowledgments, the following sentence has been added:

The authors acknowledge the work of Markus Klingenberg, who developed the therapy concept of the medical software device assessed in this research. This includes the digital implementation of the functional therapeutic approach, the device’s software-patient feedback interface, and its exercise progression algorithm.

The correction will appear in the online version of the paper on the JMIR Publications website on February 20, 2023 together with the publication of this correction notice. Because this was made after submission to PubMed, PubMed Central, and other full-text repositories, the corrected article has also been resubmitted to those repositories.

