# Supplementary material for: The Effect of an App-Based Home Exercise Program on Self-reported Pain Intensity in Unspecific and Degenerative Back Pain: Pragmatic Open-label Randomized Controlled Trial
Source: J Med Internet Res. 2022 Oct 28;24(10):e41899. doi: 10.2196/41899 (PMC9652727; doi:10.2196/41899)
Supplement: Multimedia Appendix 2 [file jmir_v24i10e41899_app2.docx]

**Supplementary Table A:** Pain score values assessed by the VNRS in the PP population at baseline, and after 2, 6, and 12 weeks of intervention.

|  | **Intervention group (n=108)** | | | | **Control group (n=105)** | | | |
| --- | --- | --- | --- | --- | --- | --- | --- | --- |
| **Time of pain assessment** | baseline | 2 weeks | 6 weeks | 12 weeks | baseline | 2 weeks | 6 weeks | 12 weeks |
| **Pain** **score (VNRS)**  *Mean (SD)* | 6.37 (1.67) | 3.68 (1.66) | 3.19 (2.17) | 2.74 (1.95) | 5.87 (1.56) | 5.51 (1.45) | 5.15 (1.79) | 4.63 (1.87) |
| **P value** intergroup treatment difference (intervention vs. control) |  | < .001 | < .001 | < .001 |  |  |  |  |
