# Supplementary material for: The Effect of an App-Based Home Exercise Program on Self-reported Pain Intensity in Unspecific and Degenerative Back Pain: Pragmatic Open-label Randomized Controlled Trial
Source: J Med Internet Res. 2022 Oct 28;24(10):e41899. doi: 10.2196/41899 (PMC9652727; doi:10.2196/41899)
Supplement: Multimedia Appendix 3 [file jmir_v24i10e41899_app3.docx]

**Supplementary Table B:** Adverse reactions reported by the study population during the 12-week intervention period

| **Reported adverse reactions reported, all adverse reactions were transient** |
| --- |
| • cervical blockage  • thigh/calve clamp  • Isolated, transient muscle clamp  • leg stiffness  • impaired hip movement  • cervical tension  • vertigo  • nausea  • strength loss  • toes fallen asleep  • movement decrease  • pain/pain increase |
